# Systematic Review of Back-Support Exoskeletons and Soft Robotic Suits

**DOI:** 10.3389/fbioe.2021.765257

**Published:** 2021-11-02

**Authors:** Athar Ali, Vigilio Fontanari, Werner Schmoelz, Sunil K. Agrawal

**Affiliations:** ^1^ Department of Industrial Engineering, University of Trento, Trento, Italy; ^2^ Department of Orthopedics and Traumatology, Medical University of Innsbruck, Innsbruck, Austria; ^3^ Robotics and Rehabilitation (ROAR) Laboratory, Department of Mechanical Engineering, Columbia University, New York, NY, United States

**Keywords:** assistive exoskeletons, back support exoskeletons, industrial exoskeletons, wearable robotics, rehabilitation robotics

## Abstract

Lower back pain and musculoskeletal injuries are serious concerns for workers subjected to physical workload and manual material handling tasks. Spine assistive exoskeletons are being developed to support the spine and distribute the spine load. This article presents a detailed up-to-date review on the back support exoskeletons by discussing their type (Active/Passive), structure (Rigid/Soft), power transmission methods, weight, maximum assistive force, battery technologies, tasks (lifting, bending, stooping work), kinematic compatibility and other important features. This article also assesses the back support exoskeletons in terms of their ability to reduce the physical load on the spine. By reviewing functional and structural characteristics, the goal is to increase communication and realization among ergonomics practitioners, developers, customers, and factory workers. The search resulted in reviewing 34 exoskeletons of which 16 were passive and 18 were active. In conclusion, back support exoskeletons have immense potential to significantly reduce the factors regarding work-related musculoskeletal injuries. However, various technical challenges and a lack of established safety standards limit the wide adaptation of exoskeletons in industry.

## Introduction

Musculoskeletal disorder (MSD) is a type of chronic physical issue resulting from repeatedly lifting heavy objects by the labor at work. It is the cause of an inordinate number of missed workdays, workers' compensation claims, skyrocketing medical bills, lost productivity, and even early retirement. Annually over 40% of the laborers in the EU agonize lower back pain due to overexertion of manual handling tasks. Most of the personnel are subjected to physical workloads due to manual handling of material, awkward body postures, and repetitive movements causing musculoskeletal injuries (Agn’s et al., 2012). In the past, different intervention solutions (instructing and educating the workers, workplace modifications, exercise, office automation, etc.) have been assessed for further prevention of Lower Back Pain (LBP) (Alemi, 2019). Since some of the intervention solutions are infeasible, expensive, longitudinal, and need necessary educative infrastructures, therefore, new intervention approaches such as wearable assistive devices have been widely explored in the past 10 years. In the modern industry, the use of robotics to improve human-robot collaboration while retaining the flexibility of humans is growing (Tucker et al., 2015; Tariq et al., 2018). The use of exoskeletons is one of the solutions for handling labor-intensive tasks. The major advantage of exoskeleton application over any type of automation system would be explicitly in dynamic environments. While automation is ideal for repetitive tasks, people are needed for a variety of tasks that require human skills, flexibility, perception, and judgment (de Looze et al., 2016).

A few literature reviews have articulated the technical aspects of the back support exoskeletons. Looze et al. review provided an outline of assistive exoskeletons which were particularly designed for industrial applications and evaluate the prospective outcome of these assistive exoskeletons in terms of physical load reduction on the wearer (de Looze et al., 2016). Toxiri et al.'s review (Toxiri et al., 2019) presents the technological advances and trends in occupational exoskeletons. However, it lacks the inclusion of other important factors such as physical load reduction, power, tasks, maximum assistive force, challenges faced by exoskeletons, and some of the new power transmission methods have not been included. In (Pérez Vidal et al., 2021; Xiloyannis et al., 2021) authors focused on challenges faced by the soft robotic suits for both upper and lower limbs including the hip joint. These reviews covered only the soft robotic suits for the hip joint, functional and structural characteristics were not discussed thoroughly. Several exoskeletons with new actuation technologies have been developed in the last couple of years and there is a need for an up-to-date review.

This article presents a systematic review of current back-support devices focusing on their type (Active/Passive), structure (Rigid/Soft), actuation technology, weight, power, battery technologies, tasks (lifting, bending, stooping work), kinematic compatibility, and other important features. This article also assesses the potential effects of back-support devices on physical load reduction of the spine. Design choices for each of these features determine the user comfort, cost-effectiveness, complexity, and biomechanical effectiveness of the resulting exoskeletons. By reviewing the corresponding factors, the aim is to develop a better understanding and enhance communication between developers, ergonomics experts, and factory laborers.

## Materials and Methods

### Search Strategy

This article presents a systematic review of the back-support exoskeleton by following PRISMA guidelines. Records are compiled by searching through several databases such as IEEE digital library, Scopus, PubMed, ASME digital library, Medline, and additional records from other sources. Naming convention in these devices is not consistent and they are mostly named based on their developer or associated research centers, regardless of their purpose and technology involved. Hence to cover maximum devices, several keywords are used which are: assistive exoskeletons, industrial exoskeletons, rehabilitation robotics, assistive technologies, wearable robotics, and back-support devices.

### Study Selection

References that report the planning phase of devices and haven’t yet taken any physical form were excluded from the review. This review is compiled of references from conferences, journals, and other commercially developed devices.

### Data Extraction/Screening Method/Focused Question

Various data variables were considered for data extraction such as actuation type, structure, force transmission methods, kinematic compatibility, tasks, power, maximum assistive force, and physical load reduction of the devices. This study aims to address following research questions (RQ):

RQ1: What are the functional and structural features of the back support exoskeletons?

RQ2: What sort of force transmission methods are available for back support devices?

RQ3: How good back support exoskeletons are in terms of physical load reduction?

RQ4: What role does kinematic compatibility play in exoskeleton design?

RQ5: What are the major challenges faced by exoskeletons regarding wide industrial adaptation?

## Results

**FIGURE 1:**
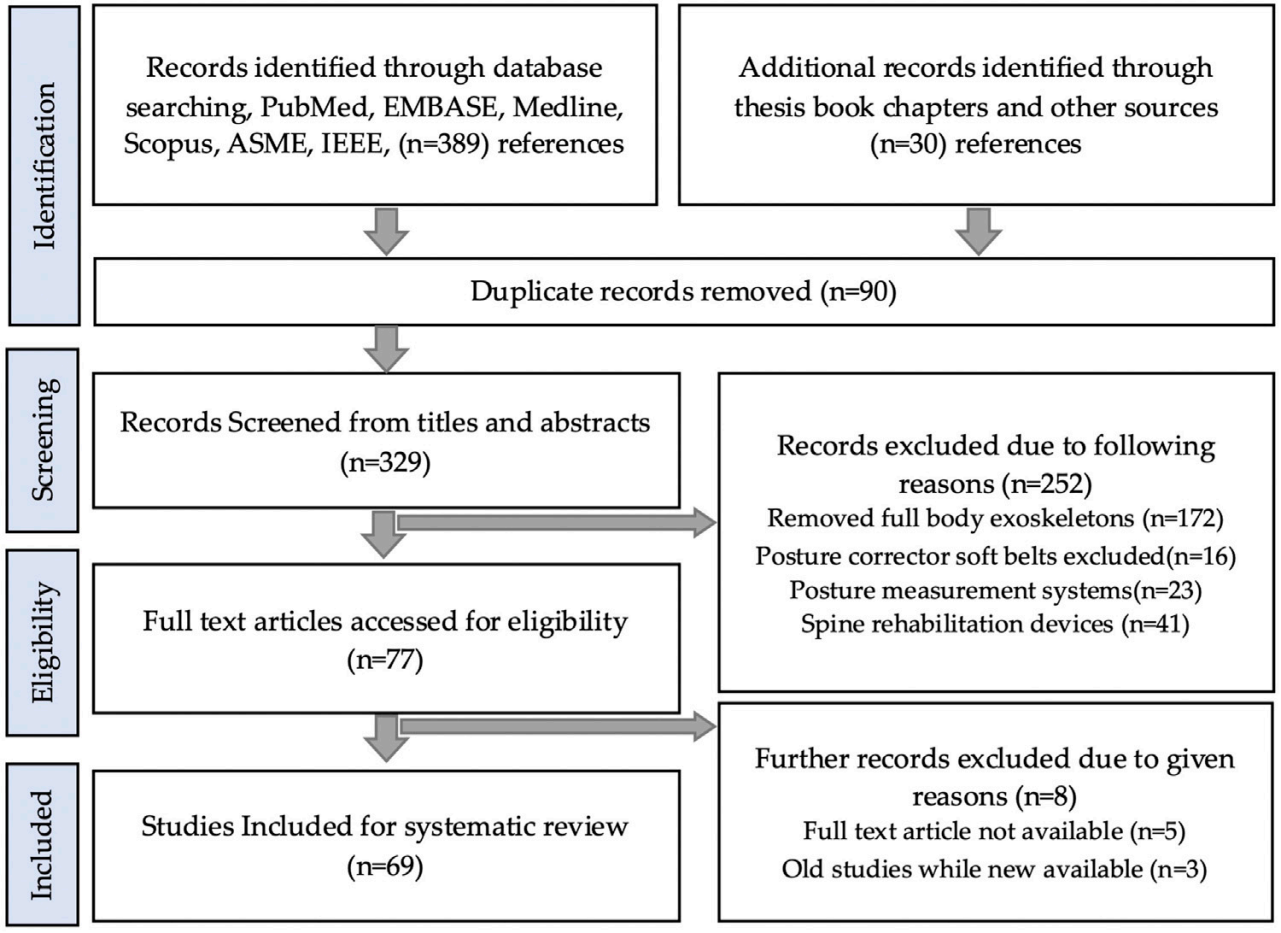
Article selection flowchart of systematic review.

Initial searches result in 419 records in total. After the elimination of the duplicate references, the records are further reduced to 329. The records were then further shortlisted by screening the titles and abstracts. This results in 77 records, which were further reduced by eliminating reference due to reasons stated in Figure 1. The final eligible 69 records were then reviewed thoroughly and incorporated into the study. This study results in 34 back-support exoskeletons of which 16 passive and 18 active exoskeletons.

## Back Support Exoskeletons

**FIGURE 2:**
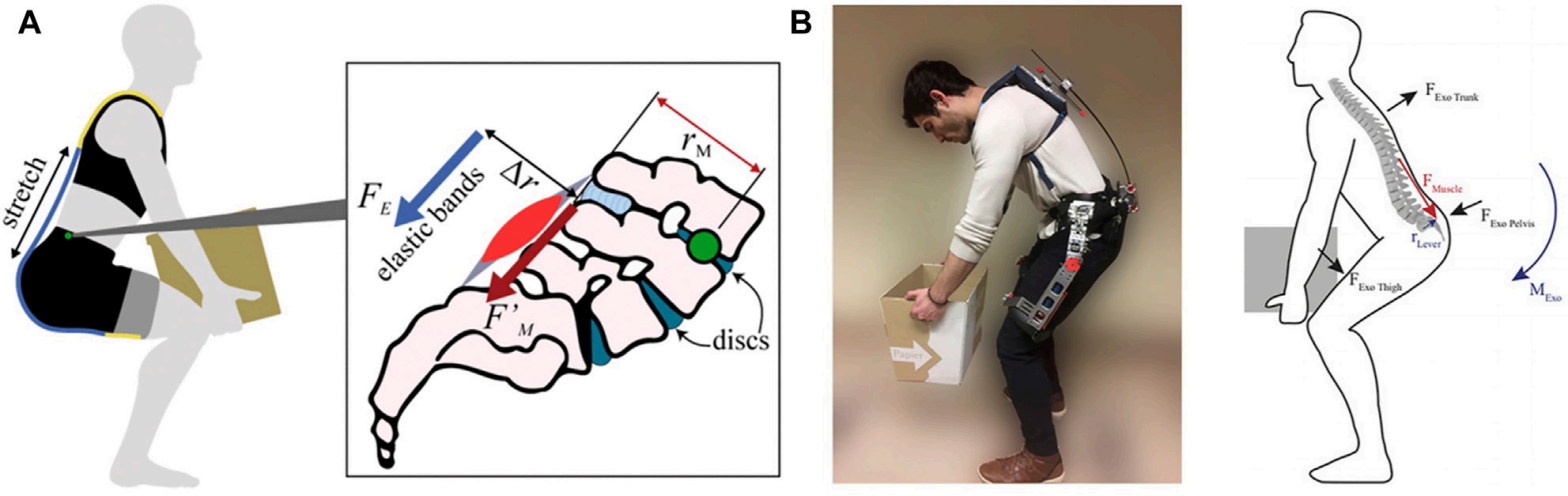
Illustration of the direction of forces applied to the user while wearing **(A)** soft and **(B)** rigid exoskeleton (Lamers et al., 2018; Näf et al., 2018).

Back-support exoskeletons are designed to offload the spine by contributing a portion of the required torque for the completion of a physical task. These devices aim to assist back extension and, in some situations, hip extension. The forces and torques could be exerted in different ways to the subject. The one key difference between current systems is the direction of the forces, which are either perpendicular or parallel to the spinal column. A force parallel to the spinal column, in addition to assisting lower back extension, also contributes to internal spine loading which is an unwanted compression on the vertebral column. However, a perpendicular force does not contribute to spinal loading (Lamers et al., 2018; Näf et al., 2018). Figure 2 illustrates the direction of forces applied on the user while wearing an exoskeleton. On the left is the soft robotic suit where arrows indicate the forces exerted parallel to the trunk and thighs. On the right, is the exoskeleton indicating forces applied perpendicular to the trunk and thighs.

In the development of back support exoskeletons, kinesiological considerations imply that the lumbosacral (L5/S1) region of the spine encounters massive mechanical loading and peak compression forces in a variety of activities. (Coenen et al., 2014). Compression forces at the L5/S1 region can reach over 5000N while just lifting a load of 15 Kg (Kingma et al., 2010). Such forces and torques are mainly due to muscle forces, required to offset the moment in the lower back caused by gravitational forces on the upper body and the load. Therefore, most back-supported devices aim at reducing compression forces in the L5-S1 region by minimizing the muscular forces necessary for the tasks. It is usually achieved by external forces which run parallel to the human back or moments which help extend the back.

**FIGURE 3:**
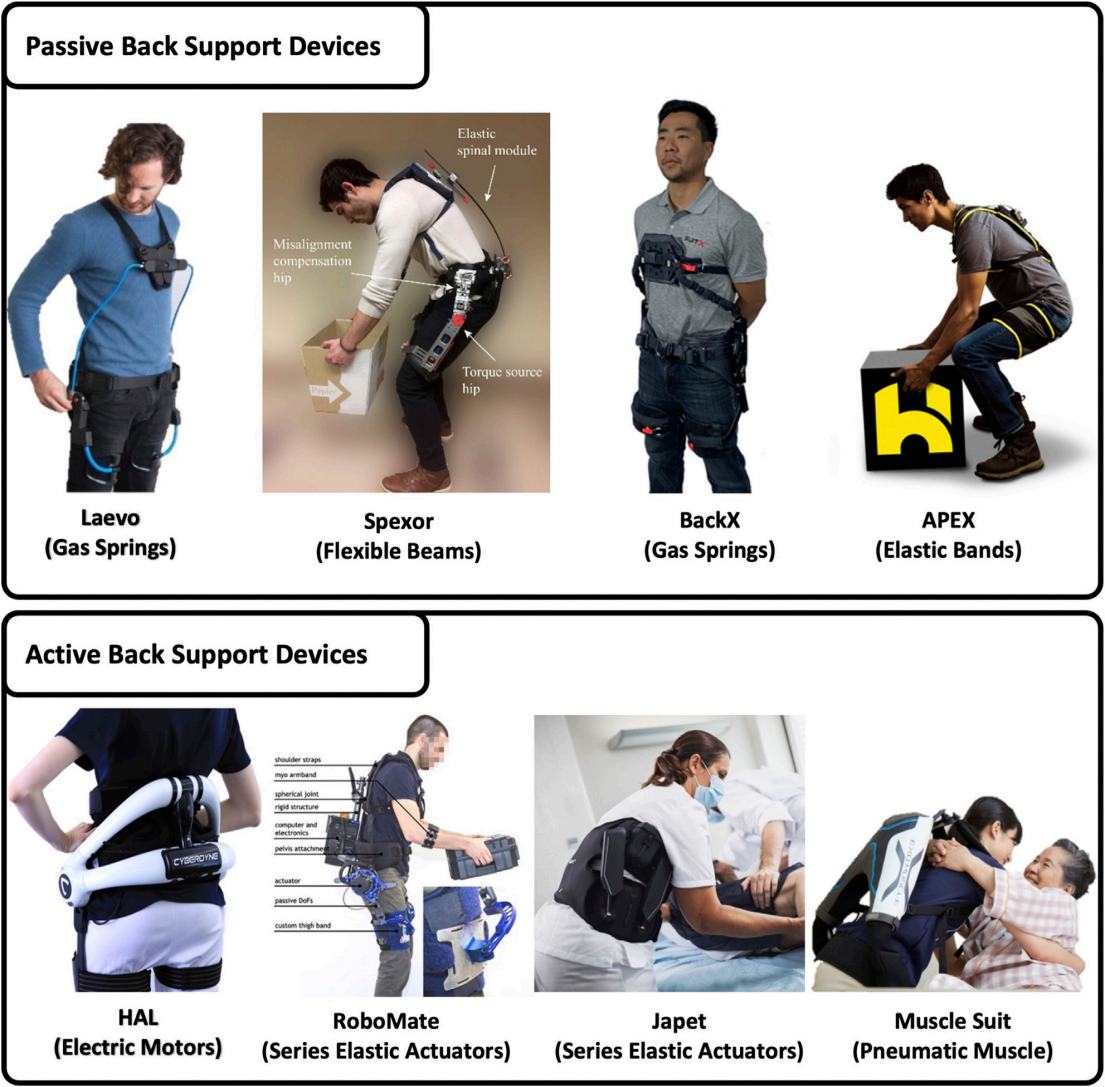
Back support devices classified based on actuation technology and structure.

As the properties of these assistive exoskeletons vary substantially with the way they are designed. They can be classified based on their actuation (active/passive), structure (rigid/soft), and the degree that the device fits or goes with the person’s anthropometry (kinematic compatibility). The selection of actuation mechanisms may define their use in different tasks. Passive exoskeletons seem more appropriate for tasks involving comparatively little assistance and less dynamic activities. On the contrary, heftier and vigorous tasks will vindicate the use of more complex active exoskeletons. The majority of back-assist devices are designed using rigid modular structures, which are hefty and bulky but produce more suitable patterns of torques and forces. A few soft robotic suits have been designed in recent years, they offer better user comfort and can be worn underneath the dress or integrated with working wear. The adoption of any assistive device will eventually depend on various factors, comprising of user acceptance, costs, and benefits associated with a specific application (Toxiri et al., 2019). Table 1 summarizes the different features of the back support exoskeletons.

**TABLE 1:** Assistive exoskeletons for the spinal column

Device	^a^Type	Structure	Features	^b^Remarks
^c^Laevo Bosch et al. (2016)	Passive (Gas Spring)	Rigid	^d^Task: L,B,R	Specially designed steel rods with a spring mechanism along with a rotating chest pad
			Weight:2.3 Kg	
			Back Muscle Stress reduction: 40%	
			Metabolic cost reduction: 17%	
^c^BackX Kazerooni et al. (2019); SuitX, (2019)	Passive (Gas Spring)	Rigid	Task: L,B,S,R (rotating)	BackX has two models S and AC. AC modes allow lateral bending and axial rotation
			Weight: 3.5 Kg (S-model)-4.5 Kg (AC-model)	
			Reduction of forces at L5/S1: 60%	
BNDR Ulrey and Fathallah, (2013b); (2013a)	Passive (Torsion Spring)	Rigid	Task: L,B,S (stoop work)	BNDR reduces torso flexion depending on the user’s weight, anthropometry, and the stiffness of the device. No kinematic compensation mechanism
			Weight: NA.	
			Load predictions were reduced: 10–31%	
WMRD Wehner et al. (2009)	Passive (Torsion Spring + Cable System)	Rigid	Task: L (Squat lifting)	Reduces the forces on the lower back by adding passive extensor restoring moment around hips which results in lowering the required extensor muscle force
			Weight: NA	
			Reduction in muscle activity: 54%	
^c^Paexo Back Ottobock, (2021)	Passive (Expander-Spring)	Rigid	MAF: (25 kgf)	Automatic clutch differentiates between walking and lifting
			Reduction in stress and strain on back: 75%	
^c^Aldak exoskeleton Systems, (2020)	Passive (Springs)	Rigid	Task: L, B	The Aldak exoskeleton is an adjustable assist-as-needed system and helps the operator lift weights with ease
			Weight: 3.5 kg	
			Weight compensation between 5 and 15 kg	
			Commercialized	
Bendezy Barrett and Fathallah, (2001)	Passive (Springs)	Rigid	Task: Stoop work	The performance was evaluated by lifting weights of 0,4,9 kg
			Weight: NA	
			Erector spinae (lumbar) activity reduction: 21%	
^c^VT-Lowe’s Simon et al. (2021); VT-Lowe’s VT-Lowe’s, (2020); Alemi et al. (2019)	Passive (Flexible Beams)	Rigid	Task: L, B	Significantly reduce the metabolic demands ∼7.9% and oxygen uptake ∼8.7% on average
			Weight: NA	
			Reduction in the mean activity of IL and LT muscles: 29.3%	
Spexor Näf et al. (2018); Baltrusch et al. (2020)	Passive (Flexible Beams + Torsion Springs)	Rigid	Task: L,B,H	Spexor incorporates compensation for misalignment for both hip and lumbosacral joints. The range of motion reduced by 10% (13◦) compared to not wearing an exoskeleton
			Weight: 6.7 Kg	
			Metabolic cost reduction: 18%	
			Muscle activity reduction: 16%	
			Reduction of forces at L5/S1: 18–25%	
PLAD (Personal lift Augmentation Device)	Passive (Elastic Band)	Soft	Task: L,B,H	PLAD does not alter the kinematics of the body and transmits forces in the form of tensions. Around 40% of the individuals reported discomfort in the knee and only 10% about shoulder discomfort
Abdoli-E et al. (2006); Abdoli-Eramaki et al. (2007); Abdoli-E and Stevenson, (2008); Whitfield et al. (2014)			Weight: NA	
			Reduction in compression and shear forces about L4-L5 23–29% and 7.9–8.5%, respectively	
			Erector Spinae T9 reduction: 14.4%	
^c^APEX exosuit Lamers et al. (2019); Herowear, (2020); Yandell et al. (2020)	Passive (Elastic Band)	Soft	Task: L	APEX has the power to reduce over 50 lbs of strain on the back with every lift Apex has a proprietary on/off the clutch to activate or deactivate the assistance
			Weight:1.54 Kg	
			Reductions in fatigue rate (ranging from 19–85%) for a subset of lumbar muscles	
Smart Suit Lite Imamura et al. (2014)	Passive (Elastic Band)	Soft	Task: B	Developed to prevent back injuries, rather than increasing the strength Used in nursing care to offload the spine while shifting the patients
			Weight: <2 Kg	
			Capable of reducing muscle activity by 24.4%	
WAD Heydari et al. (2013)	Passive (Elastic Band)	Soft	Task: HWeight: 1.5 KgReduction in the lumbar moment: 23.2–30%	Reduction for right LES, TES, LD muscles at 15 kg load and 60^0^ trunk Flexion: 23.2, 30, and 27.8%, respectively
^c^B.A Garment Lamers et al. (2018)	Passive (Elastic Band)	Soft	Task: L, Leaning,	Adequately lightweight and suitable for wearing as or underneath the clothes The performance was evaluated by lifting a weight of 24 kg
			Weight: 2 Kg	
			Reduction in leaning tasks: 23–43%	
			Reduction in lifting tasks: 14–16%		
Happy Back Barrett and Fathallah, (2001)	Passive (Elastic Band)	Semi-rigid	Task: Stoop work	The Happy Back uses bungee cords to assist during stooped work. The performance was evaluated based on lifting weights of 0Kg, 4 Kg and 9 kg
			Weight: NA	
			Reduction in erector spinae (lumbar) 23%	
Passive Spine Exoskeleton Zhang et al. (2016)	Passive (Push-pull Spring System)	Semi-rigid	Task: B	Employs a “push-pull” external assistive strategy. During flexion/extension of the spine, it applies a pushing force on the lumbar region and pulling force at the thoracic region
			Weight: 3 kg	
			Reduction at lumbar and thoracic level muscles: 24 and 54%	
RoboMate Toxiri et al. (2018a); (2018b)	Active (E) (PEA)	Rigid	Task: L, B	Control: Motion + EMG (Forearm)
			Weight: 11.6 Kg (Excluding battery and supply)	Increased the kinematic compatibility with two hinges and one ball joint
			Power: NA	
			MAF: NA		
^c^German Bionic CRAY X 2018 German Bionic, (2018)	Active (E)	Rigid	Task: L	Control: Mouthpiece, chin pad
			Weight: NA	An electrically actuated rigid exoskeleton to assist the back in lifting heavy objects
			Power: batteries 8 h	
			MAF: 25 Kgf		
^c^HAL Lumbar Support Prof, (2020)	Active (E)	Rigid	Task: L, B	Control: Motion + EMG
			Weight: 3 Kg	Charging time: 2 h
			Power: 4.5 h Batteries	14–18% reduction in forces. von Glinski et al. (2019)
			MAF:7.5 Kg	
Japet (Atlas) Bratic and Noel, (2018); Zaïri et al. (2021)	Active (E)	Rigid	Task: B	Worn like a lumbar belt and is composed of 4 micromotors to offload the spine. It follows and adjusts itself to user movements to preserve muscle activity
			Weight: 2 Kg	
			Power: 7 h Batteries	
			MAF: NA	
^c^Hyundai H-WEX Ko et al. (2018)	Active (E)	Rigid	Task: L, B	Control: Motion + Built-in algorithm for user safety. The single motor provides power to both legs. During semi-squat, the muscle activity of the Erector spinae and Gluteus Maximus reduced by 23.5 and 18.6%, and for stoop work, it reduces to 10.5 and 15.8% respectively
	BLDC + Pulley system		Weight: 4.5 Kg	
			Power: Li-Po batteries	
			MAT: 90 Nm	
			Operating voltage: 48 V	
^c^ATOUN Inc, A. ATOUN, (2020)	Active(E) Servo motors	Rigid	Task: L, B, C	Control: Motion Automatic mode (Assist, walk and break) switching based on body movement Individual left and right leg assistance control
			Weight: 4.5 Kg	
			Power: Batteries 4 h	
			MAF: 10 Kgf		
^c^APO (HuMan EU) Giovacchini et al. (2015)	Active (E) SEA	Rigid	Task: L, B	Control: Motion (State-based)
			Weight: 6.5 Kg	Powered active pelvis orthosis (APO) was developed to assist the spine in gait and other tasks
			Power: NA	
			MAF: NA		
Lower back robotic exoskeleton Zhang and Huang, (2018)	Active (E)	Rigid	Task: L, B	Control: Motion 4DOF for symmetric and asymmetric lifting Powered HAA and HFE to provide 100 Nm torque Lower back muscle fatigue decreased by 8–73%
	(SEA) + Clutch		Weight: 11.2 Kg excluding power		
			Power: NA		
			MAF: NA	
Waist Power-Assist Yu et al. (2015)	Active (E) RBE series motors	Rigid	Task: L	Control: Motion
			Weight: 8 Kg	137.42 Nm assistive torque
			Power: Li-Po batteries	With a novel clutch design
			MAF: NA	
SIAT waist EXO Yong et al. (2019); Ji et al. (2020)	Active (E) Servo motors	Rigid	Task: B, L	The average integrated electromyography reductions for LES, LD, and TES were 34.0, 24.1, and 33.9% respectively
			Weight:5 Kg	
			Power: 4 h Batteries	
			MAF: 28N	
^c^AWN-03 Panasonic Activelink, (2020)	Active (E) AC motors	Rigid	Task: L, BWeight: 6 kg (excluding battery and harness)Power: 8 h, 48 V lithium-ion batteryMAF: 15 Kgf (2 motors)	Assist mode: 3 types (lifting, holding a middle posture, assist off walking)
^c^Exo Muscle Suit Takamitsu Aida et al. (2009); Muramatsu et al. (2011)	Active (P) Mckibben PAMs	Rigid	Task: L, H	Muscle power decreases up to 69% for the elbow, 31% for the shoulder, and 37% for the waist is ascertained. It has also a passive model with a hand pump to activate the pneumatic muscle
			Weight: 5.8 Kg (reduced 2019 from 9.2 Kg)	
			Power: Gas supply	
			MAF:25.5 Kgf	
Hip Joint Exoskeleton Seong et al. (2020)	Active (E) BLDC based TSA	Rigid	Task: L	Systematically designed twisted string actuation-based hip joint
			Weight: 6 KG	
			MAF: 10 Kgf	
Soft Power Suit Yao et al. (2019)	Active (E) Maxon motors based TSA	Soft	Task: L, B	Significant muscle activation reduction for static bending (50.2–54.0%) and dynamic lifting (21.4–25.2%)
			Weight: 2.4 KG (without batteries)	
			MAF:5Kgf	
Waist assist suit AB-Wear Inose et al. (2017)	Active (P) Straight fiber-type PAMs	Soft	Task: L	Control: External operator to control In comparison with the typical McKibben PAMs, straight fiber-type has 3 times higher maximum contraction force and 1.5 times higher contraction rate
			Weight: 2.9 kg (without power)	
			Power: Pneumatic supply	
			MAF: NA	
Spine-Inspired Continuum Soft Exo Yang et al. (2019)	Active (E)	Soft	Task: L (stoop work)	Disc compression force reduction: 37%; Disc shear force reduction: 40%; Average LES muscle force reduction: 30%
			Weight:<1 KG	
			Power: NA	
			MAF: NA	
WSAD Luo and Yu, (2013)	Active (E) (Servo motors + Tension bands)	Soft	Task: L, B	Control: Motion Muscle activities were reduced by 47, 9, and 28% for LES, RA, and LD respectively
			Weight: <1 Kg	
			Power: Two cell lithium batteries	
			MAF: NA		
^c^Superflex, Seismic Powered Clothing Myseismic powered clothing, (2021)	Active (E) Muscular actuators	Soft	Task: B, W	It is not limited to a medical or an industrial setting and it includes but extends beyond sporting devices
			Power: NA	
			MAF: NA	

a Type: SEA, Series Elastic Actuator; PEA, Parallel-elastic actuator; E, Electric; P, Pneumatic; PAMs, Pneumatic Artificial Muscles; BLDC, Brushless DC motor; TSA, Twisted String Actuation

b Remarks: LES, Lumbar Erector Spinae; RA, Rectus Abdominis; LD, Latissimus Dorsi

c Commercial devices

d Tasks: B, bending; C, carrying; L, lifting; H, holding; R, rotating; W, walking

### Actuation and Structure

Based on actuation, back support exoskeletons can be catogorized as active or passive. Active assistive exoskeletons are driven by electric motors, pneumatic muscles, or hydraulic actuators, whereas the passive assistive exoskeletons often comprise cheaper mechanisms such as metal or gas springs, elastic elements, etc. These actuation or power transmission mechanisms define the structure (rigid/soft) of the exoskeletons.

#### Passive Power Transmission Devices

Passive devices such as Laevo (Bosch et al., 2016), BackX (SuitX, 2019), WMRD (Wehner et al., 2009), BNDR (Ulrey and Fathallah, 2013b; 2013a), Bendezy (Barrett and Fathallah, 2001), and Aldak (Systems, 2020), store the energy gathered by the motion of a person in springs and utilize this energy as needed to sustain a posture or assist a motion. These devices are typically rigid and to enable the motion in the sagittal plane they have in common one or more actuated hip joints. Moreover, a modular structure extends from the hip joint to the thigh, and the trunk. Devices such as VT-lowe’s (VT-Lowe’s VT-Lowe’s, 2020; Alemi et al., 2019) and Spexor (Baltrusch et al., 2020) store energy in flexible beams, this energy may support the individual to keep that posture while lifting the object.

Passive wearable devices are designed to provide maximum strength and offload the spine. Another design factor to consider in such devices is bulkiness. The devices may support lifting heavier items but with a bulky device, the user may experience discomfort. Devices such as Spexor and BackX weighing from 3.5 to 6.7 kg are heavier compared to soft suit-like devices such as Smart Suit Lite (Imamura et al., 2014), WAD (Heydari et al., 2013), B.A Garment (Lamers et al., 2018), APEX (Herowear, 2020) which weighs under 2 kg. Although, these exoskeletons are lightweight and can be worn underneath the clothes their lack of range of motion still hinders their way for wide adaptability.

#### Active Power Transmission Devices

Active devices such as RoboMate (Sposito et al., 2020), HAL Lumbar Support (Prof, 2020; von Glinski et al., 2019), AWN03 (Activelink, 2020), ATOUN(Inc, A. ATOUN, 2020), and H-WEX (Ko et al., 2018), have a higher potential in reducing the lower back loads. Both the lower body and the spinal column could benefit from a significant reduction in loading. Thus, active exoskeletons have better potential to substantially alleviate the major concerns related to musculoskeletal injuries at work.

Safety has always been one of the major concerns in human-robot interaction and it is directly related to actuation technology. Therefore, compliant actuators such as series elastic actuators (SEAs) and parallel elastic actuators (PEAs) are widely adopted because they offer a variety of advantages over conventional rigid actuators. ExMS-1 (Exodynamics ExMS-1, 2020) was developed intending to prevent back pain from progressing into severe spinal injury. It offers customizable, automatic back support without limiting the mobility using SEAs. A disk decompression device developed by Atlas Japet (Bratic and Noel, 2018) particularly helps in alleviating the back pain due to herniated disk. It is designed to relieve the pain by extending the spinal column at the lumbar region with four SEAs. Japet follows the user’s movements and adjusts itself to minimize the muscle activity in the lumbar region. These compliant actuators not only increase adaptability by deforming into various shapes but also enhance user safety by providing highly precise force control [21]. RoboMate (Sposito et al., 2020), Lower Back Robotic Exoskeleton (Zhang and Huang, 2018), and APO HuMan (Giovacchini et al., 2015) are other exoskeletons that incorporate SEAs/PEAs as actuation mechanisms.

Reducing the metabolic cost and weight of the device is one of the key challenges faced by the exoskeleton developers. An actuation mechanism that consumes less power and has a compact lightweight design would be ideal for wearable applications. One such actuation system is the twisted string actuator (TSA). TSA converts the rotation of the motor into linear motion with help of twisted strings coupled to its shaft. One can generate a higher pulling force with low-power motors. This combination results in mechanically simple, low power, and lightweight actuators (Gaponov et al., 2017). A twisted string actuators based power suit for lower back support was developed by (Yao et al., 2019). However, the authors have implemented TSAs as linear actuators in an exoskeleton design without taking the TSA characteristics into account or optimizing the hardware structure of the device by optimizing actuator size. Seong et al. systematically designed a TSA-based hip-joint exoskeleton by calculating actuator stroke and torque required for lifting tasks (Seong et al., 2020). The device has an anthropomorphic design for back support in which strings were employed along the wearer’s back. This results in significant weight reduction and enhances kinematic compatibility of the active hip joint exoskeleton. Despite the low power consumption, high force density, lightweight and compact design, TSAs have a short life cycle and low bandwidth.

Current active spinal assistive exoskeletons use motors or pneumatic actuators. These actuators are bulky and not very human interactive. Using variable stiffness mechanisms to develop the exoskeletons has huge potential. Layer jamming-based structures are emerging with a new set of possibilities among the variable stiffness mechanisms (Narang et al., 2018; Wang et al., 2019). Layer jamming structures have certain key characteristics such as lightweight, high resisting force, compactness, and fast reaction time. These structures have the capability of shape locking, which can help in reducing the metabolic cost of the device. Layer jamming structures can be fabricated using 3D printing which makes them ideal for wearable applications. Choi et al. suggested that these structures can be useful in the development of back support exoskeletons (Choi et al., 2019). However, these structures still need to get matured to be employed in the exoskeletons.

Regarding force transmission, the development of passive devices is relatively less costly, lightweight, and simpler to implement in comparison to active devices. Active devices on the other hand are more versatile and have the potential to be employed in a variety of scenarios.

### Kinematic Compatibility

**FIGURE 4:**
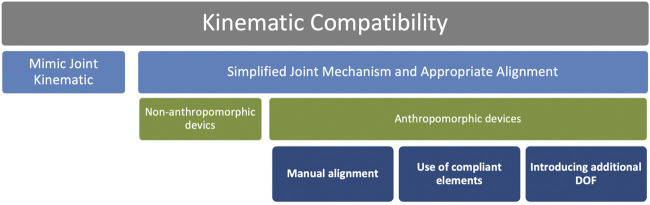
Misalignment compensation strategies.

A key element, that differentiates assistive exoskeletons, is the extent of alignment of the device’s kinematic structure with the user body. Misaligned joints can reduce comfort and generate undesirable torques and forces of up to 1.5 Nm and 230 N, respectively (Schiele, 2009). Since the perfect alignment of a device structure is difficult, rather than attempting to align the device structure with the body, devices are often designed to compensate for misalignment (Junius et al., 2017, 2018). One can achieve kinematic compatibility by mimicking the kinematics of the joints or by using simplified joint structures to ensure better alignment between the axes of rotation. It is often difficult to fully mimic the joint kinematics because the exact position of the internal rotation axis of the human joint must be replicated in an external mechanical structure. Therefore, exactly mimicking the full joint kinematics is not often practiced. Simplified mechanisms that don’t require the exact location of the joint axis of rotation are simpler to implement. These structures prevent the relative motion between the exoskeleton and the human body. Non-anthropomorphic devices usually have multiple unaligned joints, and therefore, experience several drawbacks compared to the anthropomorphic exoskeletons. Anthropomorphic devices can be aligned manually, through compliant elements, or by introducing additional degrees of freedom. Implementation of any of these alignment methods is known as misalignment compensation. Figure 4 shows the misalignment compensation strategies.

A certain level of misalignment is permissible and can be compensated by bringing more degrees of freedom to an exoskeleton. Moreover, appropriate compensation of misalignment precludes relative motion between the exoskeleton and the body, thus obliquely enhances comfort (Schiele and Van Der Helm, 2006). Introducing additional degrees of freedom will help in different lifting styles as they affect the moments required in the lumbosacral region while performing a task. Depending on lifting conditions, moments and forces at the lumbosacral region can vary significantly during lifting or stoop work. Muscle Suit (Muramatsu et al., 2013) incorporates an additional joint in the back next to the hip joint to provide an additional degree of freedom in the sagittal plane. Even though some of the devices possess a higher range of motion, they do not support the variation in different lifting styles or adopt their kinematics to compensate for the misalignment.

One must also consider the kinematic compatibility in the transverse and coronal planes while designing the exoskeleton for back support. The laevo exoskeleton features a chest pad with rotational elements to achieve somewhat differential transmission. RoboMate exoskeleton relatively incorporates the misalignment compensation in a more elaborated way, comprising of one ball and two hinge joints. A similar compensation mechanism is employed for the trunk (Junius et al., 2018). The placement of the three compensatory joints differs in the Spexor design, positioned slightly above the flexion-extension joint (Baltrusch et al., 2020). This allows the additional fitting and brings the pelvis connection closer to the body, preventing collisions of the device with the leg which might happen in higher abduction angles for the robomate exoskeleton. The Bending Non-Demand Return (BNDR) exoskeleton does not incorporate any mechanism to achieve kinematic compatibility apart from the hip joints comprising of torsional springs (Ulrey and Fathallah, 2013a). It is not evident whether any misalignment compensation is incorporated for the hip joint of the WMRD exoskeleton (Wehner et al., 2009).

Industrial exoskeletons used for the assistance of lifting and bending tasks generally include a powered flexion-extension hip joint, which moves only in the sagittal plane. They are unable to provide assistance in the frontal plane. To overcome this, SuitX developed two different models (“S” and “AC”) of the BackX exoskeleton for lumbar support (SuitX, 2019). BackX model ‘S’ and BNDR are quite similar in design, however, model “S” possess an additional abduction/adduction joint. The “AC” model of BackX has an additional joint in the back alongside the abduction/adduction joint, which allows lateral bending. A rotational joint positioned at the top of the structure incorporates axial rotation in the transverse plane as well.

Soft robotic suits have been developed and optimized for years to enhance comfort and kinematic compatibility. These devices are lightweight and mostly passive. The Personal Lift Augmentation Device (PLAD) was one of the earliest soft back-support exoskeletons, which uses tension in elastic bands to transmit the forces (Abdoli-Eramaki et al., 2007). Although soft robotic suits offer lesser reductions of biomechanical joint loading, they are comfortable and offer fewer restrictions to the user’s movements compared to rigid devices. Several passive soft devices have been developed such as Smart Suit Light (SSL) (Imamura et al., 2014), B.A garment (Lamers et al., 2018), APEX (Herowear, 2020), WAD (Heydari et al., 2013), the Passive Spine Exoskeleton (Zhang et al., 2016), and Power Assist Wear (Cho et al., 2016) which use elastic bands to transmit forces. While active exo-suits such as the waist assist suit AB-Wear (Inose et al., 2017), WSAD (Luo and Yu, 2013), and Superflex (Myseismic powered clothing, XXXX) use pneumatic or electric actuation to assist the spine.

Presence of rigid structures in exoskeletons alter the lifting technique due to misalignment between exoskeleton and human joints resulting in altered joint kinematics (Abdoli-Eramaki et al., 2007). Unlike rigid exoskeletons, soft robotic suits do not alter the kinematics of the body because no weight-bearing structure exits parallel to the wearer’s body and forces are transmitted in the form of tensions only (Näf et al., 2018). Although the absence of rigid mechanical structures significantly improves kinematic compatibility, these suits still face challenges in a range of motion (Abdoli-Eramaki et al., 2007).

### Physical Load Reductions in Back Support Devices

**FIGURE 5:**
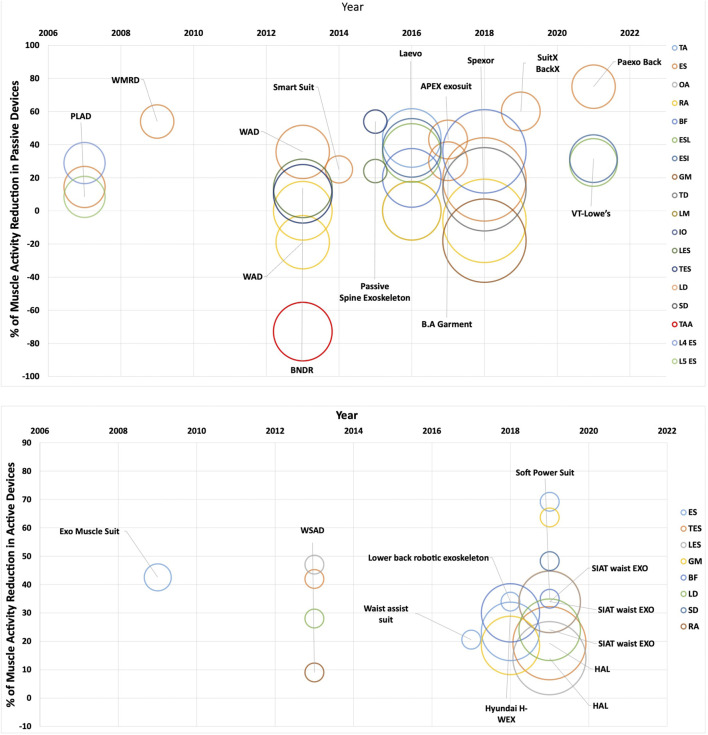
Studies reporting the change in muscular effort, resulting from wearing active and passive exoskeletons that assists the movement of the back. Color represents the investigated muscles; size represents the sample size varying between 1 and 36. Acronyms: Erector Spinae (ES), Erector Spinae iliocostalis (ESI), Erector Spinae Longissimus (ESL), Trapezius pars Ascendens (TA), Biceps Femoris (BF), Obliquus External Abdominis (OA), Rectus Abdominis (RA), Gastrocnemius Medialis (GM), Trapezius Descendens (TD), Lumbar Erector Spinae (LES), Thoracic Erector Spinae (TES), Latissimus Dorsi (LD), Semitendinosus (SD, Tibialis Anterior Activity (TAA).

The studies reporting the effects of back support exoskeletons on spine loading rely on a set of outcome measures. A metric used frequently is the rate of muscular activity reduction. Which is the outcome from wearing the device, measured by the myoelectric activity at the targeted muscles. Figure 5 shows a graphical representation of muscular reduction for specific activities reported in the studies, with an emphasis on the number of participants and their muscle activity during the task. Sample size varies from a minimum of 1 to a maximum of 36 and represented by the radius of the circles. It is noticeable from the Figure 5 that more studies have been carried out on passive devices as compared to active devices. Because active devices are mainly developed by industries and limited data is published on the physical load reductions. The circles in the positive scale of the horizontal axis represent muscles with reduced muscular activity. The circles in the negative scale of the horizontal axis represent increased muscle activity, which is mainly reported for leg muscles while wearing the devices.

#### Passive Back-Support Devices

Passive devices are designed to offload the lumbosacral (L5/S1) joint and seem to be very effective for both static holding and dynamic lifting tasks. The type of actuator and structure play a significant role in the reduction of muscle activities. The passive back support devices can be divided based on their type of assistance and rigidity. This section is categorized based on the type of assistive technology used in passive devices and their impacts on muscle activity reduction.

##### Spring-Actuated Devices

The impact of spring-actuated devices on muscle activity reductions is discussed in this section. Torque is generated between the torso and the thigh linkages by springs. This torque exerts a force on the user, compensating for the torque created by the user’s torso weight. The effect of the static holding task was investigated for the device Laevo which uses gas springs for assistance. The studies showed positive effects on lowering muscle activity during static trunk activity by 35–38%, also reduced discomfort in hip extensor activity. However, the discomfort was reported in the chest region (Bosch et al., 2016). BackX also uses gas springs as a torque generator. It showed a significant improvement in the reduction of muscle force by 60% during static stoop activity at the L5/S1 location. The study indicated that workers using exoskeletons for longer periods may experience a loss of functionality, as forward bending posture is an extremely constrained maneuver (Kazerooni et al., 2019; SuitX, 2019).

BNDR uses torsional springs to reduce compression and shear forces for stooped posture. Reductions of 13.5 and 12.1% were observed in compression and shear forces at L5/S1. This results in the reduction of load at spine tissues and ultimately reduces the risk of injury (Ulrey and Fathallah, 2013b; 2013a). A Passive Spine Exoskeleton utilizes a push-pull external assistive strategy during flexion/extension of the spine. Significant muscle reduction activity was reported at lumbar and thoracic muscles by 24 and 54% respectively (Zhang et al., 2016). The highest muscle activity reduction for passive back-support devices is reported for the Paexo Back. The device was able to minimize shoulder strain without raising lower back strain. Paexo exhibited a 75% decrease in muscle activation in the erector spine (Ottobock, 2021).

All the devices discussed in this section are spring actuated with rigid structures except Passive Spine Exoskeleton which has a semi-rigid structure. Rigid structures are reported to raise issues such as an increase in leg muscle activity, increase in muscle deconditioning, and discomfort. For a device like the Laevo, an overextended position of knees during the holding task was observed which can lead to health risks if the exoskeleton is used for longer periods.

##### Flexible Beams Actuated Devices

The spring-actuated exoskeletons can only assist in flexion/extension movements while limiting motion in other planes. To increase the range of motion in other planes, a concept for actuation consisting of multiple continuous carbon fiber beams was introduced. Spexor used flexible beams running parallel to the spine to allow a higher range of motion. It was able to reduce the torque requirements with compensation of misalignments for both hip and lumbosacral joints. Compared to other passive devices, only a moderate reduction in the back muscle forces (18–25%) was observed, while abdominal muscle forces increased. This may be due to the dynamic activity during the trials rather than the static (Baltrusch et al., 2020).

VT-Lowe’s utilizes carbon fiber-based flexible beams running in parallel to the spine up to the back of the thigh. It significantly reduced activity in the spinal muscles (29.3%) during symmetric and asymmetric lifting (Alemi et al., 2019; Simon et al., 2021.). However, moderate activity in abdominal muscles was also noticed, which can be beneficial as it stabilizes the trunk.

##### Elastic Band Actuated Devices

The majority of passive devices utilize elastic components as an external power generator positioned next to the erector spinae and thigh muscles. These elastic components transmit a portion of the spine’s forces and moments to the shoulders, pelvic girdle, and knees. As the upper body weight is decreased during lifting tasks, the elastic elements store energy, which is then released during the upward phase, decreasing the energy demand on back muscles. PLAD is a soft wearable device that uses elastic bands to assist the spine. A reduction in compression and shear forces by 14–29% was observed during various tasks. However, 40% of the individuals participating in the trial reported discomfort while wearing the device (Abdoli-E et al., 2006; Abdoli-Eramaki et al., 2007).

APEX exoskeleton incorporates a proprietary auto clutch with elastic bands to activate or deactivate the assistance. A significant reduction was observed in lumbar muscle activity and fatigue rate by 43% and 19–85% respectively (Lamers et al., 2019; Herowear, 2020; Yandell et al., 2020). WAD, another soft wearable passive device driven by the elastic band can provide a reduction in muscle forces at L5/S1 region by 23.2–30% (Heydari et al., 2013). During the study, higher acceptance was reported among the participants due to the lower weight. Similarly, B.A Garment, a soft wearable suit used elastic bands which run along the back, coupling the upper and lower-body interfaces. It reduces erector spinal muscle activity for leaning and lifting tasks by 23–43% and 14–16% respectively (Lamers et al., 2018). The device is lightweight and suitable for wearing underneath clothes.

Happy Back, a semi-rigid passive suit utilizes bungee cords to assist the motion. It offers a reduction in the erector spinae muscle activity by 23% (Barrett and Fathallah, 2001). Unlike other devices built for gaining strength, Smart Suit Lite was designed to prevent injuries and is utilized in nursing care. It has reported a reduction in muscle activity by 24.4% (Imamura et al., 2014).

Reduced back muscles activity may be accompanied by increased activity of other muscles, depending on the lifting method. An increase in Tibialis Anterior Activity (TAA) was reported for BNDR. The fact that, in both, static holding and dynamic lifting activities, external forces exerted by the device must be countered to maintain balance might explain the increase in leg muscle activation. In prolonged lifting and lowering work, increased leg muscle activity could significantly increase the metabolic demands and oxygen uptake. However, for VT-Lowe’s, the reduction of ∼7.9% metabolic demands and ∼8.7% oxygen uptake was reported.

#### Active Back-Support Devices

Active wearable devices are potentially more versatile as compared to passive devices. Their capabilities are dependent on the activation type and the utilized strategy to support the lumbosacral joint. Most of the devices are powered by electric motors and a few using pneumatic actuators.

The majority of the active back support devices are rigid and use electric motors as a mode of actuation. Several studies have been carried out focusing on the reduction of muscular activities for static and dynamic activities of these devices. HAL, a hybrid assistive limb was developed to support muscles in the lumbar region during repetitive lifting tasks. A significant amount of reduction was observed in the myoelectric activity of the thoracic and lumbar erector muscles by 19.3 and 14% respectively (von Glinski et al., 2019).

Hyundai H-WEX is another rigid exoskeleton that uses electric motors to assist the spine. The reduction in muscle activity of Hyundai H-WEX was observed to be 23.5, 18.6, and 30% for erector spine, gastrocnemius medialis, and biceps femoris (Ko et al., 2018). A similar prototype Lower-back robotic exoskeleton showed approximately 34% reduction in erector spine muscles in a study (Zhang and Huang, 2018).

The easy-to-wear SIAT waist exoskeleton is capable of reducing muscular activities around lumbar and thoracic spinal muscles by 33.9, 34, and 4.1% for TES, LES, and LD muscles respectively (Yong et al., 2019). During long-term lifting activity, the exoskeleton is reported to decrease stress and lumbar muscle strain. In the updated model SIAT-WEXv2, a 48% reduction of muscle activity of Lumbar erector spinae was achieved (Ji et al., 2020).

The Exo Muscle Suit, an active pressure-driven rigid active device uses artificial McKibben pneumatic artificial muscles (PAMs). The suit is supporting mainly the erector spine muscle group and is reported to reduce muscular activity by 42.6% (Takamitsu Aida et al., 2009; Muramatsu et al., 2011). The waist assist AB-Wear suit has a soft active driven using pneumatic actuator with a flexible flat spring structure designed to withstand compressive forces. The investigation reported muscle reduction of the erector spine by 20.6% (Inose et al., 2017). When lifting heavier objects, the trial results show that using the device can minimize muscle fatigue in the erector spinae and latissimus dorsi.

Another soft active device (WSAD) uses tension bands driven by servo motors was developed mainly for stooped postures. It reduced muscle activity of TES, LES, LD, and RA by 42, 47, 28, and 9%, respectively (Luo and Yu, 2013).

Maximum reductions of muscle activity for active back-support devices were observed for Soft Power Suit. The device is equipped with twisted string actuators, making it lightweight and capable of generating high forces in a linear direction. The muscle activity reduction measured for the erector spine, biceps femoris, gastrocnemius medialis, and semitendinosus was 69.2, 35, 63.6, and 48.25% respectively (Yao et al., 2019).

To summarize, both passive and active exoskeletons, help in muscle activity reduction at the lumbosacral (L5/S1) area, making any physical task simpler to accomplish. In this analysis, a passive exoskeleton called Paexo was shown to have the highest reduction in muscle activity. This might be because the experiment only investigated one posture. Further experiments including dynamic tasks would give a more comprehensive perspective of the muscle activity decrease for PAEXO. An active device (Soft Power Suit) on the other hand, allows for considerable decreases in muscular activity while executing a dynamic task. In terms of their impact on physical load, these studies suggest that they all can reduce muscular activity in the lower back for varying tasks (e.g., lifting and static bending). Another potential challenge many active devices face is the bulkiness of the device itself, mainly due to the weight of actuators and batteries. However, there are devices such as Soft Power suit, Waist assist suit, Spine inspired continuum soft Exo, WSAD, Japet (Atlas), and HAL which weigh under 3 kg. A device such as the Lower back robotic exoskeleton that weighs approximately 11.2 Kg excluding batteries may prove to be discomforting in the long run.

## Exoskeleton Adaptation Challenges

Passive exoskeletons are generally user-friendly, cost-effective, lightweight (1.5–3 Kg), and provide the intended range of motion. These aspects have caused passive exoskeletons to gain immense interest among ergonomics, human factors, robotics, and biomechanics researchers as well as captains of industry as a potential solution to prevent musculoskeletal back injuries. Few issues have come into the light regarding the potentially negative effects related to higher leg muscle activity, muscle deconditioning, and high levels of discomfort. On the contrary, new active exoskeletons have emerged in the market (Hyundai H-WEX5 and ATOUN Model A4), as industries have invested significantly in this area. Even though active exoskeletons have more design complexities, they have biomechanical advantages compared to passive devices because of their intrinsic versatility.

Several factors have been identified in previous studies to be responsible for lesser adaptability of the back support exoskeletons: kinematic incompatibility (Barrett and Fathallah, 2001), loss of range of motion (Abdoli-E et al., 2006; Baltrusch et al., 2018), discomfort (Abdoli-E et al., 2006; Muramatsu et al., 2013; Bosch et al., 2016), excessive force application (Abdoli-E et al., 2006), not easy to use (Barrett and Fathallah, 2001), long donning times (Junius et al., 2017), absence of weight-support functionality in soft robotic suits and lack of flexibility to be employed in a range of real-world scenarios (Bosch et al., 2016). Furthermore, there are concerns regarding the adoption of orthoses and assistive exoskeletons for an extended period of time, because they might lead to trunk muscle deconditioning. Therefore, it is essential to either restrict the time to use them or combine them with strengthening activities. Exoskeletons utilized in the industry for back support may be subject to the same situation and suggestion.

The real effect on potentially preventing back injury occurrence, yet to be evaluated, as till today substantial technical challenges and inadequate safety standards hinder the large-scale implementation in workplaces.

## Conclusion

This article comprises a systematic review of existing back-support devices in three distinct functional and structural features which are 1) actuation and structure, 2) kinematic compatibility, and 3) Reduction of muscular activities. Design choices for each of these features govern the comfort, biomechanical effectiveness, complexity, and cost-effectiveness of the resulting exoskeletons.

In terms of actuation, we hypothesize that passive exoskeletons are less complex, less expensive, and more lightweight than active exoskeletons. Active devices, on the other hand, offer a greater potential for versatility and hence a larger range of applications. Open technological challenges for active exoskeletons include optimization of the control to utilize their versatility to broaden their potential impact (Toxiri et al., 2018b). Soft robotic suits are lighter and less obstructive to movement than rigid exoskeletons, but they reduce biomechanical joint loading to a lesser extent than active ones. In terms of kinematic compatibility, soft back-support devices, do not change the body’s kinematics and simply transmit forces in the form of tensions. However, one of their drawbacks is that they are usually limiting the range of motion.

Back support exoskeletons have the potential to significantly minimize the underlying causes that lead to work-related musculoskeletal pathologies. However, the real impact on possibly lowering injury prevalence has yet to be shown, as large-scale adoption in workplaces has been hampered by major technological hurdles and a lack of specified safety requirements.
